# Well‐designed and properly conducted surgical clinical trials: Randomized control trials and big database analyses

**DOI:** 10.1002/ags3.12595

**Published:** 2022-07-09

**Authors:** Ichiro Takemasa

**Affiliations:** ^1^ Department of Surgery, Surgical Oncology and Science Sapporo Medical University Hokkaido Japan

In this issue of the *Annals of Gastroenterological Surgery,* three different study designs in clinical research in the field of colorectal cancer surgery provided intriguing results. Watanabe et al revealed safety, efficacy, and operability of an absorbable adhesion barrier in primary rectal cancer surgery with randomized controlled trial (RCT),^1^ Ogawa et al reported evaluation of clinical outcomes for colorectal cancer presenting as an oncologic emergency with propensity‐score matching (PSM),^2^ and Yamamoto et al evaluated the factors on the incidence of adhesive small bowel obstruction with a large‐scale study using a national inpatient database.^3^ The goal of clinical research is to obtain medically useful evidence through the collection of data on human subjects. To this end, it is important to minimize bias inherent in the actual clinical data and maximize the accuracy of the analyses by identifying potential sources of the bias as much as possible.

Generally, to impact the results, clinical research must have three goals: clarity, comparability, and generalizability. Ensuring clarity means minimizing bias of the random error and improving precision of the trials, and a larger sample size is the most effective way to achieve this goal. A larger sample size increases statistical accuracy and provides more definitive results. However, it is often difficult to enlarge the sample size due to ethical considerations, since clinical research involves human subjects and therefore the number of cases should be as minimal as possible. In other words, it is desirable to calculate the sample size based on the research hypothesis to be investigated in advance, and to conduct clinical research using only a sufficient number of cases. Improving comparability means minimizing bias between groups with similar background information, and randomization and/or blinding are very effective strategies for this purpose. In a typical clinical study there exists the possibility of large gaps both in number and quality between the population and the subpopulation of interest. The assessment of generalizability is to determine whether the conclusions can be extrapolated to the population beyond this gap, that is external validity, and it is useful to set eligibility and exclusion criteria and to perform subgroup analyses. The value of clinical statistics lies in the estimation of the population from the sample of interest, and a study design that eliminates bias as much as possible can be deemed to have a high level of evidence. The primary reasons for conducting multicenter clinical trials are for short‐term patient enrollment and to assess the generalizability of conclusions regarding selection bias. First, study designs of clinical research can be divided into two main categories: observational studies and interventional ones. Observational studies are conducted by observing medical records of individuals or groups as they are and analyzing the data, without any intended direct intervention, and contain cross‐sectional studies in which subjects are observed only once and longitudinal studies in which subjects are observed two or more times. Longitudinal studies are further divided into prospective cohort studies (level of evidence: IIb) and retrospective case–control studies (level of evidence: IIIb). Intervention studies are designed to provide research‐oriented interventions such as surgery to subjects and include parallel‐group studies in which subjects are divided into two or more groups and the outcomes of different interventions are compared, as well as cross‐over studies which compare the outcome of two or more interventions on the same subject at different times. In intervention studies, prospective RCT (level of evidence: Ib) can reduce bias due to covariates. Fisher’s three principles of experimental design, which aim to reduce errors, also mention the importance of randomization along with replication and local control.^4^ Since randomization is not possible in observational studies, however, PSM method is used to reduce the bias caused by covariates. PSM is considered to allow analysis of data from observational study as in RCT (pseudo‐randomized analysis), and since the level of evidence is treated as similar to that of RCT, many medical papers using PSM have been published in recent years.

Precision medicine is an attempt to accurately predict individual differences and to provide precise medical care by analyzing medical big data such as genome and other biomolecular information. Expectations are growing for this approach due to technological advances such as the significant progress in information technology, the evolution of artificial intelligence related to the utilization of big data, and the development of data science methodologies. In order to understand the current status of surgical care, big database analyses by the national clinical database (NCD), which covers more than 95% of surgeries in Japan, have been applied for practical use, and are expected to provide effective treatment selection and cost reduction. While well‐designed and properly conducted RCTs provide strong evidence, it is important to note that the results may deviate from clinical practice (real world) due to the limited population. Regarding the usefulness of RCTs versus observational studies using big databases, it has been reported that the results of the latter were comparable to those of RCTs by comparing primary endpoints.^5^ Thus, a high level of evidence can be expected by selecting a study design suitable for validating the research hypothesis and determining the number of cases to be analyzed, after incorporating the clinical questions (CQs) into the research questions (RQs).

## DISCLOSURE

Conflicts of Interest: The author declares no conflicts of interest for this article.
